# Dystonia with MPH/Risperidone Combined Therapy for ADHD

**DOI:** 10.15844/pedneurbriefs-30-1-6

**Published:** 2016-01

**Authors:** J. Gordon Millichap, Michelle M. Yee

**Affiliations:** 1 Division of Neurology, Ann & Robert H. Lurie Children's Hospital of Chicago, Chicago, IL; 2 Departments of Pediatrics and Neurology, Northwestern University Feinberg School of Medicine, Chicago, IL

**Keywords:** Risperidone, Methylphenidate, ADHD, Dystonia, Dyskinesia

## Abstract

Investigators from Child Neurology and Pediatrics, University of Texas Health Science Center, Houston, report extrapyramidal symptoms in a 13-year-old boy with a psychiatric history of schizophrenia, bipolar disorder, ADHD, and autism, responsive to combination risperidone, oxcarbazepine, and MPH.

Investigators from Child Neurology and Pediatrics, University of Texas Health Science Center, Houston, report extrapyramidal symptoms in a 13-year-old boy with a psychiatric history of schizophrenia, bipolar disorder, ADHD, and autism, responsive to combination risperidone, oxcarbazepine, and MPH. Risperidone was started at age 4 for aggressive behavior and titrated to 1.5 mg. twice daily. Because of significant weight gain, risperidone was tapered and, at a dose of 0.5 mg twice daily, he developed severe painful cervical dystonia and dyskinesia consisting of involuntary movements of the upper extremities, and uncontrollable sense of restlessness and agitation. Treatment with quetiapine, diphenhydramine, benztropine, clonidine, or lorazepam was ineffective and symptoms worsened. Risperidone was reinstated at 0.5 mg twice daily and increased to his previous dose of 1.5 mg twice daily within 3 days, after which the dystonia, akathisia, and tardive dyskinesia resolved. The authors consider this the first case of acute onset of extrapyramidal symptoms as a result of discontinuation of risperidone during combination therapy with MPH in a child. [1]

COMMENTARY. Although pediatric neurologists do not generally prescribe the antipsychotic risperidone as primary treatment for ADHD, patients taking risperidone are sometimes referred to neurology from psychiatry for a second opinion regarding refractory symptoms or untoward side-effects. The addition of a psychostimulant, methylphenidate (MPH) is a frequent recommendation and, in our experience, using conservative dosages, extrapyramidal side effects have not occurred. The present report of extrapyramidal symptoms during discontinuation of risperidone in a child taking combination RIS/MPH therapy we consider unusual and worthy of further study.

A Pubmed search in the past two years uncovered five reports of dyskinesia associated with combination RIS/MPH therapy prescribed for ADHD and comorbid disorders. An analysis of 44 cases treated in psychiatry where children received either MPH (n=28) or risperidone (n=16) as primary treatment and the majority combination treatment, symptoms of ADHD and conduct disorder were benefited, and only one case of dyskinesia occurred that resolved with the discontinuation of treatment [2]. MPH/risperidone therapy was considered particularly effective in ADHD with conduct disorder. An acute dystonic reaction in an adolescent followed the abrupt discontinuation of MPH from a combination drug regimen risperidone/MPH; the patient experienced acute dystonia on 3 occasions when he forgot to take his MPH [3]. An acute and transient dyskinesia occurred on starting long-acting MPH in a 7-year-old boy who had recently stopped taking risperidone [4]. A further report concerns three children with ADHD who developed severe hyperactivity and agitation on starting MPH after discontinuing risperidone [5]. The adverse reaction resolved after withdrawal of MPH, and following a drug-free interval, MPH was re-administered without adverse effect. In treating ADHD with the drug combination risperidone/MPH, particular care is advised when switching, starting, or discontinuing either treatment, and particularly when changing MPH. Conservative dosages are often better than mega, and to festina-lente is advised in dosage increments.
